# Book Reviews

**Published:** 1904-06

**Authors:** 


### BOOK REVIEWS.

Howe's Handbook of Parliamentary Usage. Arranged for the instant use of legislative and mass meetings, clubs and fraternal orders, teachers, students, etc. By Frank William Howe. New York: Hinds & Noble. (Price, 50 cents.)

Usually, when a physician is called to the chair in a public meeting he takes it with no little embarrassment because of his ignorance of parliamentary usage. Every physician should acquaint himself sufficiently with the simpler rules of legislation to preside with dignity, and to facilitate the prompt transaction of business. This is the best pocket-size digest of the subject we have seen and should be carried by every person likely to be called to the chair; indeed, it may be said that all members should be as well versed in parliamentary usage as the chair itself, hence all should study it. It is inexpensive, convenient and useful; moreover, it is authoritative, being compiled from the best manuals,—Cushing, Robert, Reed, and Palmer. It is properly named—the instantaneous arbitrator.

---

Transactions of the American Dermatological Association. Twenty-seventh annual meeting held at Washington, D. C., May 12-14, 1903. Charles J. White, M.D., Secretary. New York: The Grafton Press.

The transactions of this society always possess special interest for the dermatologist, because they contain the latest thought of the best observers in this important branch of medicine; and, again, because rare cases of disease of the skin are described and illustrated,—conditions that often are not met more than once or twice in a lifetime of such experience.

In the present volume Dr. G. W. Wende, of Buffalo, presents a paper of this nature entitled, Sarcomatosis cutis, with two illustrations, the first showing the lesions in situ on the right leg of the patient, and the second being a microphotograph of a section of the growth, showing round cell infiltration around bloodvessels and glands, as seen when the lesions first appeared.

The volume contains several other papers detailing rare skin lesions, some of which are illustrated. The work done by the society and herein recorded indicates a splendid organisation of trained men, working together for the advancement of an important branch of medical science.

---

Biographic Clinics. Volume II. The Origin of the Ill Health of George Eliot, George Henry Lewes, Wagner, Parkham, Jane Welch Carlyle, Spencer, Whittier, Margaret Fuller Ossoli and Nietzsche. By George M. Gould, M.D., editor of American Medicine. Duodecimo, pp. 392. Philadelphia: P. Blakiston's Son & Co. 1904.

The author of this unique, theoretical study into the causes of the ill health of these distinguished personages, seems dissatisfied with the treatment his first volume received at the hands of the reviewers. He says that scarcely one summarised a clear and satisfactory statement of the thesis, facts, and arguments of the book. He thinks a great mistake is made in underestimating the importance of eyestrain. We were of the opinion that this subject was one that was receiving due attention by the profession, thanks to the efforts of our ophthalmologic specialists of which there is, at least, not a scarcity.

We have no disposition to quarrel with our distinguished friend in his contention, nor do we feel like going into a lengthy analysis of his "clinics." We prefer to make suitable reference to them in these columns, merely inviting attention to their entertaining character and let each, for himself, judge of their intrinsic worth as contributions to medical science.

---

Mother and Daughter. Advice to the Maiden, Wife and Mother. By C. A. Button, M.D., Holland, N. Y. Holland Medical Company, Holland, N. Y. (Price, $1.00.)

A manual of this kind for household reading and guidance is sometimes of value, especially when placed in the hands of intelligent persons. This book may be read with benefit by every mother intelligent enough to appreciate the importance of proper hygiene, or who can properly apply the simple directions given for many conditions needing attention pending the arrival of a physician.

The principle underlying the book from cover to cover is that the first thing to do in case of illness is to send for a competent physician; meanwhile, in certain conditions some of the advice herein given may be applied. Despite its defects in style and faulty construction here and there, it is a household manual of considerable value.

---

PREVENTIVE MEDICINE. Two Prize Essays: The General Principles of Preventive Medicine, by W. WAYNE BABCOCK, M.D.; the Medical Inspection of Schools, a Problem in Preventive Medicine, by LEWIS S. SOMERS, M.D. Published by The Maltine Company, Brooklyn. 1903.

These essays, it will be remembered, were selected out of 209 presented in competition for prizes offered two years ago by The Maltine Company. The committee met in Buffalo to make the awards, which were promulgated October 18, 1902. The following paragraph from the report is of sufficient interest to reproduce: "In submitting this report the committee congratulates you (The Maltine Company) upon the wide spread interest which you have aroused in the very important subject of preventive medicine, and it congratulates the medical profession and the public upon the great good that will follow the publication of the valuable addition to literature thus evoked by your enterprise." Signed, Daniel Lewis, (New York); Charles A. L. Reed, (Cincinnati); John Edwin Rhodes, (Chicago).

It is sufficient for us to add that these essays are published in one volume for gratuitous distribution, by The Maltine Company, and may be obtained upon application to the publishers.

---

THE COMPLETE MEDICAL POCKET-FORMULARY AND PHYSICIAN'S VADE-MECUM. Containing upwards of 2,500 prescriptions, collected from the practice of physicians and surgeons of experience, American and foreign. Also a special list of new drugs, with dosage, etc. By J. C. WILSON, A.M., M.D., physician to the German Hospital, Philadelphia. Third revised edition. Philadelphia: J. B. Lippincott Company. (Price, $1.75; thumb indexed, $2.)

The preparation of this reference manual containing about twenty-six hundred prescriptions represents a surprising amount of work. Each formula must be given with the greatest care, lest error creep in which would be capable of great harm. The proof reading of such fine type taxes the eye and the patience to an extreme degree. It is as complete as it is possible to make such a work and is capable of doing great good to the profession. It is useful to the junior physician whose repertory of prescriptions is necessarily somewhat limited, and to the older practitioner whose memory needs refreshing. It contains, besides, a special list of new remedies, their therapeutic applications, and dosage; table of formulæ for suppositories; formulæ for hypodermic medication; list of drugs for inhalation; poisons and their antidotes; a posological table; list of incompatibles; table of weights and measures; list of disinfectants, and numerous other useful hints.

The Treatment of Fractures: With Notes upon a Few Common Dislocations. By Chas. L. Scudder, M.D., Surgeon to the Massachusetts General Hospital. Fourth edition. Thoroughly revised, enlarged and reset. Octavo volume of 534 pages, with nearly 700 original illustrations. Philadelphia, New York, London: W. B. Saunders & Company. 1903. (Polished buckram, $5.00 net; sheep or half morocco, $6.00 net.)

Testimony as to the value of this treatise may be cited in the unexampled demand for new editions, this being the fourth in as many years. There is no branch of surgery that tests the competency of a surgeon more than the treatment of fractures. Unless one has an aptitude for this work he had better abandon it at once, or still better, never begin it. The young practitioner is always put to his mettle in dealing with broken bones. There is no treatise on the subject at the present time that will prove as helpful to him as this one; it is the next thing to the clinic itself.

The author has added many half-tone illustrations to this edition, thereby enhancing its value. The text, too, has been increased somewhat, and a chapter on a few common dislocations has been appended. Attention should be invited to the remarkable fact that this book of 534 pages contains 688 illustrations,—a proportion rarely equaled in a medical publication. Scudder has a talent for dealing with fractures and dislocations and he has displayed it to the best advantage in this superb treatise.

THE PRACTICAL MEDICINE SERIES OF YEAR BOOKS. Ten Volumes. Issued under the general editorial charge of GUSTAVUS P. HEAD, M.D., Professor of Laryngology and Rhinology in the Chicago Post-Graduate Medical School. Volume III. The Eye, Ear, Nose and Throat. Edited by Casey A. Wood, M.D., Albert H. Andrews, M.D., and Gustavus P. Head, M.D. Duodecimo, 332 pages. Chicago: The Year Book Publishers. 1903. (Price, $1.50; entire series, $5.50, payable in advance.)

The year books arrive with the same regularity as the years themselves. It seems but a short time since this series was inaugurated, yet this is the third time these subjects have been presented, and by the same editors. But little change has been made in the plan, except a reduction in price, rendered feasible we presume by increased sales. The wealth of literature in these branches as in others proves a great embarrassment to the compilers, but, it must be confessed, they have done well in selecting and condensing. It should be borne in mind that this series of year books is intended for the general practitioner in particular, the object being to give him such information as will keep him in touch with all branches of medicine. At the same time, the arrangement is such, that separate volumes may be obtained by those interested in special subjects, without the necessity of purchasing the entire set. The laryngological part of this number is of unusual interest and places in the hands of the general physician, who must needs do some of this work, a mass of helpful material. By and large, the series furnishes one of the best year books, and at a price that makes it possible to all.

---

The Blues, (Splanchnic Neurasthenia), Causes and Cure. By Albert Abrams, A.M., M.D., (Heidelberg), F.R.M.S., Consulting Physician, Denver National Hospital for Consumptives, the Mount Zion and the French Hospitals, San Francisco. Duodecimo, pp. 240. Illustrated. New York: E. B. Treat & Co. 1904. (Price, $1.50.)

The title of this book though bizzare is impressive; it is unscientific but "fetching." The author offers as a reason for the term "blues," the statement that "it appeals with cogent significance to the sufferer,"—apparently the person for whom the book is really intended, it being written upon lines to interest the laity.

The author has called attention to many conditions of importance and has restated many facts of known value and significance in relation to neurasthenia; especially are his remarks regarding the sexual hypochondriac of excellent quality. But in all the essential points upon which he places emphasis there is not much that is novel. The most that can be said is that Abrams has given new names or new locations to some old conditions that are pretty well understood.

It is a readable book, written in attractive style and will do every physician good who will take the pains to read it with care. The method of treatment by automassage is at least not harmful and may serve to amuse if it does not absolutely cure.

---

The Self-Cure of Consumption Without Medicine. With a Chapter on the Prevention of Consumption and Other Diseases. By Chas. H. Stanley Davis, M.D., Meriden, Conn. Pages, 176. New York: E. B. Treat & Co. 1904. (Price, 75 cents.)

Much may be learned by the unprofessional person from reading this book. It tells many things that every person should know, and it teaches some things even to the professional reader. The author is quite right when he asserts, rather as a refrain pervading the entire brochure than as a dogmatic asseveration, that the successful management of the consumptive depends upon the patient, upon the care he takes of himself and the thoroughness with which he carries out the intelligent directions of a competent medical adviser, rather than upon the ingestion of drugs, many of which prove harmful instead of beneficial. Cough mixtures are especially to be condemned.

The directions given in regard to proper breathing are of great value and may well be heeded by every person; correct breathing is an essential to good health, even for those who are in no special danger of consumption. We repeat, this book contains much that will benefit both profession and laity.

---

PROGRESSIVE MEDICINE. Volume I, March, 1904. A Quarterly Digest of Advances, Discoveries and Improvements in the Medical and Surgical Sciences. Edited by HOBART AMORY HARE, M.D., Professor of Therapeutics and Materia Medica in the Jefferson Medical College of Philadelphia. Octavo, 337 pages, 7 illustrations. Philadelphia and New York: Lea Brothers & Co. (Per annum, in four cloth-bound volumes, $9.00; in paper binding, $6.00, carriage paid to any address.)

The volume for March, 1904, deals with surgery of the head, neck and thorax, by Charles H. Frazier; infectious diseases, including acute rheumatism, croupous pneumonia, and influenza, by Robert B. Preble; diseases of children, by Floyd N. Crandall; laryngology and rhinology, by Charles P. Grayson; and otology, by Robert L. Randolph. This copy is bound in paper and is stamped on the front cover in large red letters, "For Review." We think these two innovations detract from the hitherto handsome appearance of this excellent periodical. The material, however, maintains the high level established by the editors and well sustained by the contributors.

---

A COMPEND OF PATHOLOGY, GENERAL AND SPECIAL. A Student's Manual in one volume. By ALFRED EDWARD THAYER, M.D., Professor of Pathology, University of Texas. Duodecimo, pages 711; 131 illustrations. Second edition. Philadelphia: P. Blakiston's Son & Co. 1903.

Two years ago Professor Thayer issued two compends on pathology, which are now merged in one book and are here presented in a handsome volume in black flexible leather, with rounded corners and gilt edges. While, manifestly, it is a book intended for the undergraduate student, it is yet one that may be studied with profit by the practitioner who needs to refresh his memory on old points, or to familiarise himself with newer ones. The method adopted by the author in presenting his subject is simple, direct, and entertaining; his science is well grounded and his language forceful without being diffuse. His instructions for tests, cultures, and blood examinations such as Widal's, are clear and pointed. On the whole, it is a most satisfactory manual and will increase in popularity through the present style of issue.

---

BLAKISTON'S QUIZ COMPENDS. Compend of Diseases of the Nose, Throat and Ear. By JOHN JOHNSON KYLE, B.S., M.D., Lecturer on Otology, Rhinology and Laryngology in the Medical College of Indiana, Indianapolis. Pages, 280, with 85 illustrations. Philadelphia: P. Blakiston's Son & Co. 1903. (Price, 80 cents.)

The fundamentals of laryngology are set forth in the form of a compend in this book, and in a manner to meet the requirements of the student approaching examination day, as well as the junior practitioner, who must of necessity treat the ear, nose, and throat in a limited capacity. It presents in this fashion the best condensed thought of the most accepted authorities on this subject, and may be appealed to with safety and satisfaction. In brief, there is no better epitomised laryngology than the Kyle compend.

---

TRANSACTIONS OF THE NEW YORK ACADEMY OF MEDICINE. Semi-centennial Celebration, 1896-1901, including the Semi-centennial Celebration Addresses, January 29, 1897, and List of Papers read at Stated Meetings of the Academy. Printed for the Academy. New York. 1903.

The addresses in this book are of special interest and deserve preservation in permanent form. That of the Hon. Grover Cleveland, then President of the United States, is published in facsimile. One of the most interesting papers in the volume is the memorial address on Joseph O'Dwyer, by W. P. Northrup. It tells the story of intubation from its inception to its perfection, all through the struggles of the inventor from obscurity to fame, the death of his lovely wife, and the final breakdown of the talented genius of the laryngeal tube. It is a touching story of disappointments, trials and triumphs, that cannot fail to reach the heart.

The New York Academy of Medicine is rich in its memories, rich in its traditions, rich in its accumulated medical lore; the present volume is evidence of the truthfulness of these statements. May its shadow never grow less!

---

TRANSACTIONS OF THE AMERICAN GYNECOLOGICAL SOCIETY. Volume 28. For the year 1903. Twenty-eighth annual meeting held at Washington, D. C., May 12-14, 1903. J. Riddle Goffee, M.D., Secretary. Philadelphia: William J. Dornan, Printer.

An examination of this volume discloses the melancholy fact that many of the veterans of this great society are passing away. Herein are recorded the memorials of John Byrne, Gaillard Thomas, and Edward Jenks,—nature's noblemen all. Most of the papers printed in this book are written by newer members, though a few veterans are still in the field and some of them occupy its center. The president, Janvrin, discourses most ably and clearly upon the surgical treatment of cancer. He advises hysterectomy, either vaginal or abdominal, as the only operation when the diagnosis is early made. He approves, however, of other operations in later diagnoses, for cosmetic and other reasons.

The newer members,—Coe, Vineberg, Harris, Charles P. Noble, Manton, Burrage, Goffe, and Clark,—have presented a valuable group of papers; and the veterans, Johnson, Palmer, Mann, as well as Janvrin, have not lost one whit of the old vim, as shown by their papers and discussions.

When the American Gynecological Society starts out to make an interesting book in which it records its annual proceedings it is a great success. When, however, it permits a coterie of its membership to endeavor to persuade a certain group of the Fellows of the American Association of Obstetricians and Gynecologists to join its ranks, it cannot be regarded as having reached its highest function. It is quite time such tactics should cease; it has never won over the men who have been the real object of its crusade and it never will. Those men have nothing but contempt for the men who engage in it, though the very highest respect for those other dignified, erudite, and distinguished men who constitute the majority.

Diverted for the moment from our original purpose, we return to it with the object of once more accentuating the value of the work which this society puts out each year, and of remarking in particular that the present volume is quite up to the high ideal of the leading members of this scientific body of professional workers.

---

TRANSACTIONS OF THE STATE MEDICAL ASSOCIATION OF TEXAS. Thirty-fifth Annual Session held at San Antonio, Texas, April 28 to May 1, 1903. H. A. West, M.D., Secretary.

The medical profession of the state of Texas has been distinguished for many years as maintaining a high standard of efficiency and proficiency, due in a large measure if not entirely to the influence of the association from which this book emanates. For many years these transactions were edited by Dr. Hamilton Atchison West, who died December 30, 1903, this volume being almost his last work,—entirely so in a literary sense, we believe. To Dr. West, more than to any other man, does the Texas profession owe its present status, and to him is it indebted for its *esprit de corps,* which is of a very high order. The present volume is quite the largest and best ever issued by the association, and indicates a future of great moment if the organisation progresses, as no doubt it will, on the lines already laid down. It is to be hoped that the association will not be betrayed by the *ignis fatuus* of state journalism. To abandon its splendid volume of annual transaction for the weak and imperfect monthly state journal, would be to exchange a strong individuality for a mediocre position in the publication of its transactions.

